# Providing culturally safe care to Indigenous people living with diabetes: Identifying barriers and enablers from different perspectives

**DOI:** 10.1111/hex.13168

**Published:** 2020-12-22

**Authors:** Marie‐Claude Tremblay, Maude Bradette‐Laplante, Holly O. Witteman, Maman Joyce Dogba, Pascale Breault, Jean‐Sébastien Paquette, Emmanuelle Careau, Sandro Echaquan

**Affiliations:** ^1^ Département de médecine familiale et médecine d’urgence Faculté de médecine Université Laval Québec QC Canada; ^2^ Groupe de Médecine de famille universitaire du Nord de Lanaudière Joliette QC Canada; ^3^ Vice‐décanat à la responsabilité sociale Faculté de médecine Université Laval Québec QC Canada; ^4^ Centre Mihawoso Centre de pédiatrie sociale Manawan QC USA

**Keywords:** barriers and enablers, community‐based participatory research, cultural safety, diabetes, healthcare inequities, indigenous peoples

## Abstract

In recent years, cultural safety has been proposed as a transformative approach to health care allowing improved consideration of Indigenous patient needs, expectations, rights and identities. This community‐based participatory study aimed to identify potential barriers and enablers to cultural safety in health care provided to Atikamekw living with diabetes in Québec, Canada. Based on a qualitative descriptive design, the study uses talking circles as a data collection strategy. Three talking circles were conducted with Atikamekw living with diabetes and caregivers, as well as with health professionals of the family medicine teaching clinic providing services to the community. Two team members performed deductive thematic analysis based on key dimensions of cultural safety. Results highlight four categories of barriers and enablers to cultural safety for Atikamekw living with diabetes, related to social determinants of health (including colonialism), health services organization, language and communication, as well as Atikamekw traditional practices and cultural perspectives of health. This study is one of the few that provides concrete suggestions to address key aspects of diabetes care in a culturally respectful way. Our findings indicate that potential enablers of cultural safety reside at different (from individual to structural) levels of change. Solutions in this matter will require strong political will and policy support to ensure intervention sustainability.

**Patient or public contribution:**

Partners and patients have been involved in identifying the need for this study, framing the research question, developing the data collection tools, recruiting participants and interpreting results.

## 1 INTRODUCTION

In Canada, the prevalence of diabetes is four times higher for Indigenous adults living on reserve in comparison with the general population.^1^ The onset of this disease is rooted in historical context of colonial policies and harmful experience in residential schools, including metabolic long‐term effects of starvation and stress,^2^, ^3^ experiences of food restrictions that may promote subsequent inadequate nutrition behaviours,^2^, ^3^ food insecurity in remote regions where some Indigenous communities are located^4^ and environmental degradation of traditional food sources such as fish and maritime products.^5^

In recent years, cultural safety has been proposed as a transformative approach to health care allowing improved consideration of Indigenous patient needs, expectations, rights and identities.^6^, ^7^, ^8^, ^9^ Cultural safety aims to support Indigenous patients by dismantling colonialism currently embedded in the healthcare system. While literature on this topic is still scarce, culturally safe clinical practices are associated with higher levels of satisfaction and improved clinical outcomes for patients living with diabetes.^10^ Ensuring a culturally safe approach to diabetes care is especially important since this condition involves coordinated care and frequent encounters with the healthcare system and health professionals, who play an important role in good diabetes management. Some authors have proposed framework and principles for cultural safety in primary care,^11^ but the application of these principles in diabetes care stays complex and not wholly understood.^12^, ^13^, ^14^

As a previous step of this project, our team conducted a rapid review of the scientific literature to identify interventions that improve cultural safety for Indigenous people living with diabetes in the healthcare setting.^10^ Our review only found seven studies (including two from Canada) that highlighted cultural education, modified environment of care and integration of the Indigenous workforce as relevant strategies to improve cultural safety in diabetes care. While these results provide a good starting point, they consist in scattered strategies implemented in various settings, which are sometime far different from those in Canada. The current stage of knowledge on cultural safety in diabetes care does not allow to develop a local understanding of this issue nor to support an informed clinical practice. There is a need to identify current obstacles to cultural safety in health care for patients living with diabetes as well as relevant strategies to implement this concept in partnership with Indigenous peoples in Canada. This community‐based participatory study aimed to identify potential barriers and enablers to cultural safety in health care provided to Atikamekw living with diabetes in Québec (Canada).

### 1.1 Context of the project

Manawan is one of the three Atikamekw communities (First nation of the Anishinaabe cultural group) located in Lanaudière (Québec, Canada), about 189 km from Joliette, a city with more complete and specialized health services available. Atikamekw communities are bonded by shared cultural values, which include caring for extended family and community, respect for elders, equality between men and women, as well as a relationship of interdependence with the territory.^15^ Thanks to the continuing oral transmission from one generation to the other, 95% of Atikamekw people still speak the Atikamekw language.^15^ Although many Atikamekws are bilingual in that they also speak French (the official language of the province of Quebec), some elders and young children are unilingual, speaking Atikamekw but not French.

Like many Indigenous communities in Québec, Manawan experiences a high prevalence of chronic diseases and related complications. The prevalence of diabetes is estimated at 25.6% of Manawan adults.^16^ In cooperation with Health Canada, Manawan manages a local community health centre (Centre Masko‐Siwin), a nursing station which offers some primary care services. Other specific arrangements are in place, such as a transportation service that runs 5 days a week to transfer Manawan patients needing care to Saint‐Charles‐Borromée's hospital, Joliette. The Manawan local health centre is supported by the nearest family medicine teaching clinic, *Groupe de Médecine de famille universitaire de Saint‐Charles‐Borromée* (GMF‐U SCB), located in Joliette. In 2017, doctors from the GMF‐U SCB did three‐day rotations twice a month at the local centre in Manawan and provided on‐call support service 24/7. To improve effectiveness in answering the needs of their patients, the GMF‐U SCB implemented a patient group management system, which involves assigning patients (i.e. patients from Manawan) to a group of doctors, rather than to a specific doctor (group‐practice model).

In 2017, the GMF‐U SCB recognized challenges in following up on and meeting the needs of Indigenous patients living with diabetes. Clinicians from the GMF‐U SCB contacted the principal investigator (MCT) at Université Laval (Québec, Canada) to help improve their services and better meet their patients’ needs. They were concerned about patients’ lack of adherence to care and the difficulty to conduct follow‐ups. They suggested that these issues may be linked to cultural aspects of care for Indigenous patients.

## 2 METHODS

### 2.1 Design and approach

This study builds on a participatory approach involving a partnership between a research team, the community of Manawan, the Native friendship centre of Lanaudière and the GMF‐U SCB. Organizational and community partners have been involved in identifying the need for this study, framing the research question, developing the data collection tools, recruiting participants, and interpreting results. Based on a qualitative descriptive design, the study uses talking circles as a data collection strategy. Talking circles are frequently used to collect data in many Indigenous contexts, offering a means to collect data that encourages story‐telling and collective listening.^17^ Following this method, participants sit in a circle and are invited to speak in turn about a specific issue in a respectful and safe manner. This study is approved by the Research Ethics Board of both Université Laval (no. 2017‐205) and the CISSS de Lanaudière (no. 318‐03‐N‐14).

### 2.2 Conceptual framework

**FIGURE 1:**
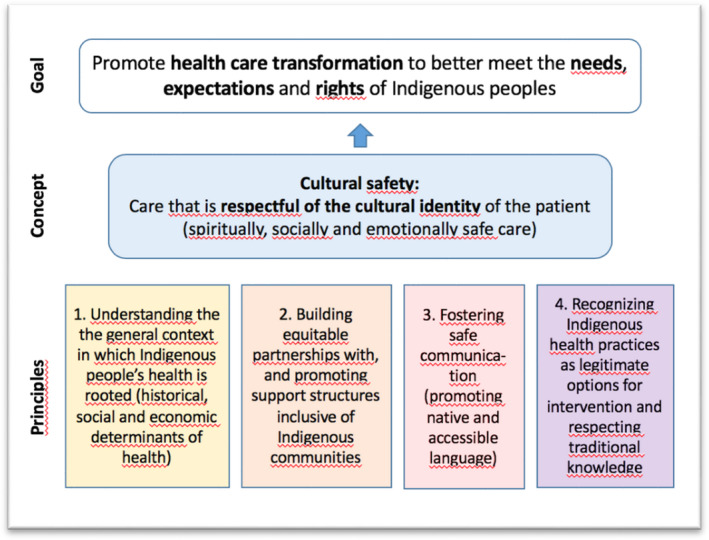
Concept of cultural safety (adapted from Smye et al.)

This study is rooted in the concept of cultural safety. Cultural safety involves an equitable partnership between patients and health professionals that enables parties to recognize, respect and nurture the unique cultural identities of Indigenous populations.^6^, ^7^, ^8^, ^9^ There is a lot of variability in the way cultural safety is conceived and applied.^12^, ^18^ Although interpretations of this concept differ,^12^ they usually share similar features. In this project, we used a definition of cultural safety that emphasizes concrete principles and different levels of application of this concept, as conceived by Smye and colleagues.^8^ According to Smye et al,^8^ cultural safety requires four key principles (Figure 1). The first principle involves health professionals understanding the general context in which Indigenous people's health is rooted. This includes considering both the influence of historical, social and economic determinants affecting health of Indigenous populations, and the devastating impact of colonialism and intergenerational trauma on the health of Indigenous peoples.^8^, ^12^, ^19^ The second principle focuses on building equitable partnerships with Indigenous communities and promoting support structures inclusive of Indigenous communities, including elders, families and health‐care professionals.^8^ The third principle requires ‘safe communication’, which involves not only promoting the patient's language as much as possible, but also using accessible language to communicate with the patient, free of technical or medical jargon.^8^ The fourth principle of cultural safety is based on the recognition of Indigenous health practices as legitimate options for intervention, and respect for Indigenous traditional knowledge.^8^, ^19^

### 2.3 Data collection

We conducted three talking circles with three different groups of participants (total of 30 participants). The groups of participants (Table 1) included Atikamekw living with diabetes and caregivers in Manawan (Group 1), Atikamekw living with diabetes and caregivers in Joliette (Group 2) and health professionals working at the GMF‐U SCB (Group 3). Participants were recruited on a voluntary basis, with help from the community health centre in Manawan (Group 1), from the Native Friendship Center in Joliette (Group 2) and the GMF‐U SCB in Joliette (Group 3). Recruitment strategies used formal invitations through email for health professionals. For Atikamekw living with diabetes (themselves or their family), recruitment built on advertising using social media and webpages of partners, community radio announcements, and direct solicitation by partners. Discussion guides were developed in partnership with two Indigenous patient partners and organizational partners of this project. Two different versions of the guide were developed, one for people with diabetes and caregivers, and the other for health professionals.

**TABLE 1:** Participants’ characteristics

Group	Gender		Relationship to diabetes		Health professionals’ roles			Total
	Women	Men	Person with diabetes	Caregiver to a person with diabetes	Physicians	Nurses	Pharmacist	
1 ‐ Atikamekw patients and caregivers from Manawan	7	7	10	4	‐	‐	‐	14
2 ‐ Atikamekw patients and caregivers in Joliette	5	1	4	2	‐	‐	‐	6
3 ‐ Health professionals working at the GMF‐U SCB	8	2	‐	‐	4	5	1	10
Total	20	10	14	6	4	5	1	30

Talking circles were led between May and June 2018. They were conducted in culturally safe places identified by partners, that is, a meeting room in a Lodge in Manawan (group 1), a room in the Native Friendship Center in Joliette (group 2) and a meeting room in the GMF‐U SCB (group 3). At their arrival, the participants were greeted by the researcher team and were invited to partake of a light (and diabetic appropriate) meal, which is a culturally appropriate way to thank Indigenous participants for their participation. Talking circles started with a land acknowledgement by the researchers, followed by a brief introduction of each person, including the researchers. The principal investigator and a member of the research team moderated the discussions. A first subject of discussion was introduced to the circles by the researchers, and the participants were invited to speak in turn while holding a talking stick that had been provided by the Native Friendship Center. Participants were invited to listen respectfully in a non‐judgemental way, and everyone was allowed to remain silent if they preferred. Talking circles ranged from 1 hour (with health professionals) to 2 hours (with Atikamekw patients and caregivers) in length. Discussions were led in French and were audio‐recorded. All participants provided written consent and received a financial compensation.

### 2.4 Data analysis

Interviews were professionally transcribed verbatim, and transcripts were verified by a research team member (MBL) for accuracy. All transcripts and project documents were analysed using NVivo 12. Two team members (MCT and MBL) performed deductive thematic analysis^20^ based on the four principles of cultural safety as initial themes. While mainly deductive, the coding process remained flexible enough to allow for current themes to be redefined or potential new themes to emerge. The two analysts (MBL and MCT) generated the initial codes and then associated codes to themes relevant to barriers and enablers of cultural safety in care. Coding differences were resolved by consensus. Partners and some project participants discussed and validated results at a deliberative dialogue workshop. Participants generally agreed with the interpretation proposed, but suggested some modifications in wording, such as using ‘family’ and ‘extended family’ instead of ‘informal caregiver’ (as initially proposed).

## 3 RESULTS

Results highlight four categories of barriers and enablers to cultural safety for Atikamekw living with diabetes (Table 2). These four categories generally mirror the four principles of cultural safety previously described. The second principle (i.e. relationship and support structures) has been reframed as structures of health service organizations, which emerged from the data as a more relevant theme.

**TABLE 2:** Barriers and enablers to cultural safety for Atikamekw living with diabetes

Social determinants of Indigenous health (including colonialism)	Health services organization	Language and communication	Traditional practices and cultural perspectives of health
Barriers			
Experiences of discrimination or racism within the health‐care system Health recommendations not adapted to Atikamekw patients’ socio‐economic situation	Difficulty considering the social organization and values of the Atikamekw community within the structure and delivery of health care A group‐practice model that complicates establishing safe therapeutic relationships Congestion in the healthcare system that makes it hard to implement a patient‐centred approach	Health professionals’ ignorance of the Atikamekw language Use of medical jargon by health professionals Health professionals’ lack of knowledge around Atikamekw social and communication codes	Health education material not suitable for Atikamekw culture or traditional practices Health professionals’ ignorance of Atikamekw cultural practices in relation to health and spiritual aspects of health
Enablers			
Education sensitizing health professionals to discrimination and racism. A systematic mechanism to handle complaints regarding racism and discrimination in healthcare organizations	Reorientation of health services to consider the social organization and cultural values of Atikamekw patients Recruitment of Indigenous health staff and professionals	Interpretation service Education for health professionals regarding basic terms in the Atikamekw language Utilization of simple language in healthcare encounters	Culturally appropriate educational material Education for health professionals regarding Atikamekw culture, values and health practices Exploring and validating patient preferences, expectations, values

### 3.1 Colonialism and social determinants of Indigenous health

#### 3.1.1 Barriers

A first barrier to culturally safe care highlighted by patients and caregivers is racism in health organizations. While not a general rule, participants of Groups 1 and 2 mentioned having personally experienced or hearing about people receiving differential treatment due to their Indigenous status, in various contexts. Such experiences include not being taken seriously by health professionals or being treated disrespectfully:

An Indigenous person who goes, for example, to the hospital to receive care, I am speaking in general and also within diabetes, often we as Indigenous person are spoken to poorly. The reception is not…it’s different. So we are not going to be considered as people. (Group 2)

Racist or differential treatment led people with diabetes to decide not to consult. For instance, one participant reported:

I speak for my daughter‐in‐law (…) Once she was evacuated there because her sugar level was too high. Then when she arrived at the emergency room, she hears the nurses say, 'The natives come here just to look for pain killers'. (…) Since then, my daughter‐in‐law has never wanted to go back when she has peaks like this (Group 1)

Another barrier to culturally safe diabetes care that relates to social determinants of health is the difficulty for health professionals to consider patients’ socio‐economic situation when providing health recommendations. It might be difficult for Indigenous patients to apply some of the advice health professionals give due to their precarious social and economic situation. For instance, healthy eating might be challenging since access to fresh and healthy foods can be an issue in the community, as noted by one patient participant:

Also family income… there are some families who are not able to pay for salads. It’s pretty expensive. Fruits even, and cheese also, it’s not a given for everybody (Group 1)

Barriers related to the mistrust instilled by colonialism were also identified by health professionals as a potential explanation for non‐compliance with their recommendations:

I often have the impression that there are things unsaid or secret, maybe not unveiled, which can create barriers in everything we tell them. We are not aware of the experiences of these people sometimes, which might perhaps explain why they do not respond to all that is asked of them. (Group 3)

#### 3.1.2 Enablers

Corresponding levers for action associated with colonialism and social determinants of health were also identified. According to participants, one enabler of cultural safety associated with this category is providing education that sensitize health professionals to discrimination and racism, and foster attitudes of openness and respect. For instance, one patient mentioned:

Like when I talked about my daughter‐in‐law earlier, I would like that there in the hospital that the staff too, the nurses, be sensitized. (…) That the staff there in the hospital stop judging. They put us all in the same package. I deplore that a lot. (Group 1)

Another potential solution brought by participants is to develop and implement a mechanism to systematically handle complaints regarding racism and discrimination in health organizations. As suggested by a patient:

When I go to a ward for treatment, the hospital is not necessarily aware of what is going on downstairs. Someone would have to meet with a manager in the hospital, so that they know what they are doing there. (…) Maybe put a service as patient protector, something like that. As an ombudsman but in the hospital for healthcare and social services. (Group 2)

Participants emphasized solutions to colonialism that went from providing education to professionals at an individual level, to changing support structures for patients in organizations.

### 3.2 Health services organization

#### 3.2.1 Barriers

A second category of barriers to culturally safe care for Indigenous living with diabetes is related to the health‐care system's organization. From participant perspectives, health services appear ill‐adapted to the social organization and values of the Atikamekw culture. Indigenous patients and caregivers notably evoked the fact that family members and caregivers were not always invited to the hospice day centre, where a lot of diabetes education is done:

Then what I find good for people living with diabetes is the day center there. You have a lot of teaching. But it would be important for the spouse to go with his spouse, because they live together (…) And sometimes it is the spouse who makes the food and then sees what is taught there. So it helps at home. (Group 1)

A health professional echoed this concern by reflecting on how individualism is embedded in the health‐care system:

We have a model of care that is very focused on the individual … at the level of confidentiality, at the level of appointments, at the level of care decisions, we look at people individually. I think that sometimes it clashes… a decision shared with a patient, is it a decision that will be shared at home with the father, the grandmother? (…) we are not used to thinking according to that. (Group 3)

Along the same lines, community social support—which is seen as essential for Atikamekw people with diabetes in crucial moments such as diagnosis or episodes of acute care—is not always facilitated in the current system. A participant remarked the ignorance of a nurse towards their cultural customs and values:

I looked after my husband when he was sick [with other people from the community] (…). There is a nurse who said 'Hey there is a big party here'. They laughed at us. (…) Then there was a woman who answered, she said 'No, ma'am, that's not it. We do this when a person is sick, we support until the end.' (Group 1)

Another barrier reported by the participants was the group‐practice model being put in place by the GMF‐U SCB. Health professionals mentioned that some patients found it hard to be followed by several doctors instead of having the opportunity to develop a trusting relationship with one. One of the health professionals reported:

Often they want to see the doctor again, then it is certainly our system that makes sure they do not necessarily see us again, and then the connection is more difficult. (…) sometimes there are some who are less comfortable with that. They prefer to see the same person again, in 3 months. (Group 3)

Finally, congestion in the health‐care system itself was mentioned as an important barrier related to health service organizations. This kind of obstacle was mostly identified by participating health professionals. Congestion was considered as inhibiting the implementation of a patient‐centred approach, which would allow to better consider the cultural diversity of the clientele.

Then we’re on the go, we’re on the go, we prescribe, we do our best, but if we had more availability we might have more time to have more in‐depth discussions, and not just do physicals. (…) Yes, we try to take the time, but when we have twenty patients and we are two hours late, we have less time to look for these barriers. (Group 3)

Establishing a culturally safe therapeutic relationship requires time to share and bonds with patients, which appears unrealistic to health professionals in the current system due to their heavy patient load.

#### 3.2.2 Enablers

Some enablers of culturally safe diabetes care identified by participants are located at the level of health services organization. Reorientation of health services would allow to better consider the social organization and cultural values of Atikamekw, for instance by favouring the involvement of family in care or by developing new models that better suit the needs of patients. A health professional remarked that a new follow‐up model started to be implemented in the community, which appears more aligned with the community's values:

[One of our doctors] tries a little bit to go to the houses, to do some follow‐up at home. This involves many things to add to all this just for notes, investigations, compensation. Sure it would be a beautiful model to implement, but it would be necessary that the rest of the machine follows in all that. (Group 3)

Some patients suggested the idea of a hospice centre exclusively reserved to Indigenous people and run by Indigenous staff:

Then when you bring (the elderly) to a center, it's even worse in there. Because they're here 24 hours a day, but you do not know what's going on in there. Then there would be that too, a need to have this here for us, a fair center for Indigenous people with Indigenous staff. (Group 2)

Another suggested potential lever for action was hiring more Indigenous health staff and professionals. For instance, a health professional mentioned that he was looking to integrating an Indigenous doctor within the team:

One solution would be to integrate an Indigenous family doctor within the team. (…) There is one [Indigenous person] who currently applies medicine (…) actually, I offered this person to come and do observation with us, integrate it (…). But perhaps [the solution] would be to support or accompany an Indigenous people who has an interest in going into medicine in their career, to help a little. Eventually, of course, if there was an Indigenous doctor who joined us, it would be really helpful to really have their vision, and then he could share how they perceive health. (Group 3)

Participants provided ideas that involved changing how care is delivered, where and by whom, in order to create safe spaces that respect Atikamekw culture and values.

### 3.3 Language and communication

#### 3.3.1 Barriers

Language and communication were consensually identified as prominent barriers to culturally safe care by all groups of participants. These barriers relate to the fact that many Atikamekw are unable to obtain services in their mother tongue (i.e. Atikamekw) while consulting outside of their community. Given the fact that many Atikamekw have no or limited abilities in French, this creates a situation where some patients (e.g. elders) cannot even communicate with their health professional:

Let's say when they are seniors who come, it’s certain they do not speak French. Then all there is when they come to their appointments is a paper marked 'follow‐up'. Follow‐up of what? Abdominal pain? But abdominal pain OK, there are several parts here. How do you want the elder to explain where they are hurting and what the symptoms are? (Group 2)

This problem was reworded by a health professional in similar terms:

Then we must not forget also that French is their second language. There is all the aspects of interpretation, there are several words that do not exist in Atikamekw that we use in French (…). (Group 3)

Medical jargon used by health professionals further exacerbates this issue, hindering mutual understanding even when patients speak French. As exemplified by one patient:

Often, I ask: can you repeat what you just said in other words, because I don’t understand. And then, most of the time, they explain in more understandable terms. Sometimes doctors are a bit like lawyers, we do not understand anything they say. They have a way of speaking. This makes sure that people who are sick, they arrive bewildered and then understand nothing. (Group 2)

Participants highlighted the use of medical jargon by health professionals as creating misinterpretations of words, undermining trust and adoption of health professionals’ recommendations. The issue of misunderstanding was also reported by health professionals. For instance, a doctor noticed that two of her patients had completely stopped their diabetes medicine after the first year, because they had not understood that this kind of medication has to be renewed after one year (Group 3).

Moreover, Atikamekw cultural codes of conduct remain unknown by health professionals. A patient mentioned that a health professional once asked him to maintain eye contact, being unaware of this behaviour being against their cultural code of conduct, as it is considered rude. Health professionals also noticed cultural differences related to communication. Atikamekw patients are seen as more silent and reserved than their general clientele, and this is perceived as a communication obstacle between them:

It is difficult to know if they are really interested. They express few words. They are often very quiet. (…) It is difficult to know what it is that they want. (Group 3)

In short, language and communication barriers were perceived as hindering mutual understanding and trust, which is fundamental to the development of a safe therapeutic relationship.

#### 3.3.2 Enablers

Enablers to culturally safe communication in health care were also identified by participants. Providing interpreter service was mentioned by a majority of participants. Another potential solution identified by many was in health professionals learning basic terms of the Atikamekw language. A professional related that she has started to use some words of Atikamekw during consultations as a way to comfort her patients:

I started to do it lately, it's really practical: it's just that I learned two words, hello and then goodbye in Atikamekw. That makes it a little touch, then I ask 'OK can you explain me how to say goodbye?' It's really a small start, but I say to myself over time, I will never be bilingual, but I will learn maybe some casual words like that. I say to myself that at the base it is just respectful of the other. (Group 3)

A final suggested strategy to enable cultural safety in communication is to promote the utilization of simple language in health‐care encounters. According to a patient, this involves ‘taking the time to explain well, to use terms that we can better understand’. (Group 2).

### 3.4 Traditional practices and cultural perspectives of health

#### 3.4.1 Barriers

A last category of barriers to culturally safe care is related to the difficulty to take into account Atikamekw traditional health practices, and cultural perspectives of well‐being. One specific obstacle is that health education material disseminated to Atikamekw living with diabetes is not relevant to Atikamekw food and lifestyle. For instance, an informal caregiver reported:

You have a panoply of medications to take, then what you are often given for your diet are pamphlets that already prepared, which are not pamphlets adapted for example to what Atikamekw eat and cook. (Group 2)

On this matter, a health professional also acknowledged:

It must also be said that our cardio‐metabolic course is not really adapted to this clientele. This is a question we asked ourselves if there was something specific to adapt to their culture. (Group 3)

A patient that had previously worked as a nurse in the community remarked that adapted material already exists, but was not really used:

We made an Atikamekw food guide, then we never see it here … When I was still working, I distributed it to the families and then brought it to the hospital. (…) But now I do not see it. (Group 1)

Another important barrier related to this category is the health professionals’ limited knowledge of traditional health practices, and cultural and spiritual concepts of disease. This kind of barrier was mostly identified by health professionals as follows:

The entire spiritual side that we no longer have, is still very present among them. (…). Then there is all the traditional therapy, the plants and all that, and then there are all the ceremonies that go with that. But we do not have access, so we do not know very much. (Group 3)

Health professionals were aware of their lack of knowledge about Atikamekw traditional practices and health concepts, which was considered an obstacle to providing culturally safe care:

Maybe also the concept of health that is different. I am not an expert but in terms of health, (…) at what point is their concept of being healthy different, and what this represents in comparison [to our concept]? If we do not agree on this already at the grassroots level, it is a little difficult to provide care that is culturally acceptable or safe if it does not meet their expectations. (Group 3)

They expressed their desire to know more but were unsure about where to start and how to apply this knowledge in practice.

#### 3.4.2 Enablers

Potential levers for action associated with traditional practices and cultural perspectives were suggested by participants. A first solution identified was in providing culturally appropriate health education material for Atikamekw living with diabetes (e.g. an adapted nutrition guide). Patients and health professionals were aware that some resources specific to Atikamekw culture already existed and they emphasized the importance of using them.

Another important strategy identified by participants was to provide education for health professionals regarding Atikamekw culture, values and health practices. A patient highlighted the value of educating people about Indigenous culture to bridge gaps:

I think people would be different in Joliette if we put more public activities to inform people. Once they invited us to downtown [to introduce our culture] … It was just to explain, but we do not often have the opportunity to do that. I think people would see something other than what they see. (Group 2)

Along the same line, health professionals expressed their interest in knowing more about Atikamekw traditional health practices:

I think we had some training but maybe if we could have more … To know that they do something special with the healer for diabetes … to know exactly what to ask them … what [cultural practices] they integrate into their everyday lives? Just having more information would eventually help us to better integrate their culture, I think. (Group 3)

Similarly, health professionals suggested exploring and validating patient preferences, values and expectations regarding health and health care during consultations. For instance, a health professional evoked the idea of an implicit initial contract, which would allow them to better focus on the patient's expectations and needs in the therapeutic relationship:

Probably we do not ask them enough about what's important to them, and what are their expectations of healthcare? Often in the clinic, we also feel that there are many people to see, so we are very focused on the treatment of the disease, [we do] a little bit of prevention. But we probably do not ask enough what they come for, what are their expectations. Maybe we could do some kind of initial contract, which would make it possible to explore that more at the beginning before entering the medical records. (Group 3)

In sum, participants emphasized the importance of being, not only more sensitive to, but also more knowledgeable of culture, values and perspectives in order to ensure diabetes care that respects Atikamekw's needs and practices.

## 4 DISCUSSION

While many patient participants mentioned generally having good health‐care experiences, these results provide clearer evidence of a service gap in appropriate care available to Atikamekw living with diabetes at clinics practising Western medicine. This study is one of the few that provides concrete suggestions to address key aspects of diabetes care in a culturally respectful way. While all barriers and enablers identified might not relate specifically to diabetes (e.g. racism and discrimination in the health‐care system, health‐care professionals’ ignorance of the Indigenous cultural perspectives), the findings highlight the importance of providing culturally appropriate education and tools, fostering simple language and offering realistic, adapted medical advice for Indigenous peoples living with diabetes. These practices might be of particular importance in the case of chronic diseases such as diabetes, which requires the patient to have both a deep understanding of the disease, an extensive personal commitment to self‐management and frequent encounters with the health‐care system. Reflecting on other ways of offering health care in culturally relevant ways (e.g. follow‐up at home, favouring family and peer support in care) is crucial to better enhance follow‐ups and coordination of care required in the context of diabetes. This might require health‐care providers to give up practice group models in place of more patient‐centred practice models that facilitate building trust and respect within the therapeutic relationship.

Our findings clearly indicate that potential enablers of cultural safety in diabetes care emerge at different levels, from individual to systemic levels of change. At the individual level, cultural safety requires improving health professionals’ knowledge of traditional practices, cultural perspectives of health and diabetes and cultural competence (i.e. possessing the skills to act while considering cultural difference). At the organizational and systemic level, it requires deep structural changes from organizations and systems with a willingness to better accommodate the diversity and needs of their clientele. While some suggestions (e.g. integrate mechanisms to handle racism and discrimination, transform health‐care models and hire more Indigenous staff) made by participants address this level of action, these solutions also require support at the policy and systemic levels to increase sustainability of cultural safety and maintain accountability.

Following this study, a deliberative dialogue workshop was organized with all stakeholders of the project to discuss the results and prioritize potential solutions to improve cultural safety in diabetes care. A total of 21 people attended the event, including Atikamekw living with diabetes, representatives of the Manawan community Council, professionals from the Native Friendship Center, GMF‐U SCB health professionals and health decision makers from the hospital and regional health authority. Before the workshop, all participants received a summary of the results in accessible language. Following the small group and plenary discussions, participants identified an innovative solution embracing many of the potential levers of action described in the results. The proposed solution involved developing and implementing a new professional role dedicated to cultural safety in health‐care organizations. This ‘cultural safety officer’ would juggle multiple tasks, including supporting and advocating for patients, training health professionals and fostering transformations of health organizations and clinical practices.

### 4.1 Validity and limitations

Results of this study must be interpreted in their context. Reliability and quality of the results were strengthened through the use of a database, a rigorous protocol and through a structured thematic analysis strategy involving two analysts. Interpretation of the results was validated by participants and stakeholders of the project through a deliberative workshop where results were presented and discussed. However, results are based on a limited number of views that are specific to the Atikamekw community and generalization to other contexts should be done with caution. Furthermore, participants may not be representative of all Atikamekws, since they were most likely to speak and understand French.

## 5 CONCLUSION

This study aimed to identify potential barriers and enablers to cultural safety in health care provided to Atikamekw living with diabetes. Results of this study offer insight about the struggles Indigenous patients face when entering health organizations, and practical ways cultural safety can be enhanced within clinical encounters and health organizations. Our findings clearly indicate that potential enablers of cultural safety reside at different levels (from individual to systemic) of change. Solutions in this matter will require strong political will and policy support to ensure intervention sustainability, as well as change of practices and norms.

## CONFLICT OF INTEREST

The authors declare no conflict of interest.

## AUTHOR CONTRIBUTIONS

This research has been designed and developed by the principal author (MCT) in collaboration with partners and co‐authors (HOW, MJD, PB, JSP, EC, SE). MCT and MBL collected the data and carried out the analysis. Results have been interpreted and discussed by partners and all authors (HOW, MJD, PB, JSP, EC, SE). MCT and MBL wrote a first version of the paper. Then, all co‐authors (HOW, MJD, PB, JSP, EC, SE) read and approved the final manuscript.

## Data Availability

The data that support the findings of this study are available on request from the corresponding author, upon agreement from community partners. The data are not publicly available due to privacy or ethical restrictions. The community of Manawan retains ownership of all data, and control over data and their use is managed by the Manawan community Council.
